# A Beneficial Role of Rooibos in Diabetes Mellitus: A Systematic Review and Meta-Analysis

**DOI:** 10.3390/molecules23040839

**Published:** 2018-04-06

**Authors:** Moe Sasaki, Nami Nishida, Masako Shimada

**Affiliations:** 1 Graduate School of Nutritional Science, Sagami Women’s University, 2-1-1 Bunkyo, Minami-ku, Sagamihara, Kanagawa 252-0383, Japan; s1771101@st.sagami-wu.ac.jp; 2 Faculty of Nutritional Science, Sagami Women’s University, 2-1-1 Bunkyo, Minami-ku, Sagamihara, Kanagawa 252-0383, Japan; ntake39928@yahoo.co.jp

**Keywords:** rooibos extracts, diabetic rodent models, blood glucose levels, meta-analysis

## Abstract

In a rapid increase in cases of diabetes mellitus worldwide, there has been interested in the use of plant-derived polyphenols as nutraceuticals to prevent the onset and progression of diabetes mellitus and its associated complications. *Aspalathus linearis*, commonly known as rooibos, is a rich source of uncommon glycosylated plant polyphenols with various critical health-promoting properties, including the prevention and treatment of diabetes mellitus (DM). This study aimed to examine these effects by meta-analyzing the current evidence in diabetic rodent models. Peer-reviewed studies written in English from two databases, PubMed and Embase, were searched up to 28 February 2018. Studies reporting blood glucose levels in diabetic rodents with and without receiving rooibos extracts or their major phenolic compounds are included. Twelve studies enrolling 88 diabetic rodents treated with rooibos extracts or their polyphenols and 85 diabetic control males reported blood glucose levels. The pooled effect size was −0.89 (95% CI: −1.44 to −0.35) with a substantial heterogeneity (*I^2^* = 67.0%). This effect was likely to be modified by type of rooibos extracts and their polyphenols and treatment period. Blood glucose levels were significantly lower in diabetic rodent models treated with the phenolic compound rich in rooibos extracts, PPAG.

## 1. Introduction

The number of individuals living with diabetes mellitus (DM) was estimated to be 425 million in 2017 and this figure is expected to reach 629 million by the year 2045 [1]. Type 2 DM (T2DM) is a chronic metabolic disorder that makes up about 90% of DM cases, and primarily occurs as a result of obesity and lack of exercise. T2DM is characterized by high blood glucose levels, insulin resistance in the muscle, liver and adipose tissues and relative deficiency of insulin secreted from the pancreas. Moreover, patients with DM often develop various complications including triopathy, dyslipidemia and cardiovascular diseases, which are the major causes of their mortality [1]. Therefore, it is critical to explore new strategies to combat DM.

In recent years, there has been a growing interest in various natural products including herbal teas and traditional Chinese medicinal or desert/semi-desert plants in prevention and treatment of diabetes due to their natural origin and relatively less side effects than pharmaceuticals [2,3,4,5,6,7,8]. *Aspalathus linearis*, rooibos, is usually grown in the Cederberg, a small mountainous area in the Western Cape province region of South Africa [9]. Compared with green and black teas rooibos tea is a caffeine-free and low-tannin beverage that contains various minerals and polyphenols, including dihydrochalcones (aspalathin (ASP) and nothofagin), phenylpropenoids (phenylpyruvic acid-2-*O*-glucoside (PPAG)), flavones (isoorientin and orientin) and flavonols (quercetin-3-*O*-robinobioside). Because of its unique properties, rooibos tea has gained popularity around the globe, particularly among people such as expectant and nursing mothers who are encouraged to avoid caffeinated beverages.

Two forms of rooibos teas are available: fermented and unoxidized “green” tea. The fermented rooibos extracts (FREs) contain high levels of major phenolic compounds, isoorientin (mean 11.8 ± 2.6 mg/g dry powder, 25.2% *w/w*), orientin (7.9 ± 0.7 mg/g, 16.9%), quercetin-3-*O*-robinobioside (7.4 ± 2.9 mg/g, 15.8%), PPAG (6.7 ± 0.4 mg/g, 14.3%) and ASP (3.7 ± 0.0 mg/g, 8.1%) [10,11]. Other flavonoids including nothofagin, vitexin, isovitexin, hyperoside, rutin and isoquercitrin are also found at a concentration of less than 3 mg/g dry powder of FREs or GREs. Water-based green rooibos extracts (GREs) contain about three times higher levels of total phenolic compounds than FREs (GREs, mean 160.6 ± 22.7 vs. FREs, 46.7 ± 8.0 mg/g dry powder). Quantitative composition analysis of GREs showed that ASP is the major flavonoid (106.2 ± 16.5 mg/g, 66.1% *w/w*), followed by isoorientin (13.4 ± 2.4 mg/g, 8.3%), nothofagin (11.7 ± 2.5 mg/g, 7.3%), orientin (10.6 ± 1.6 mg/g, 6.6%), queretin-3-*O*-robinobiside (6.4 ± 0.6 mg/g, 4.0%) and PPAG (2.9 ± 0.4 mg/g, 1.8%) [11,12,13]. ASP (C_12_H_24_O_11_, 452.4 Da) and nothofagin (C_21_H_24_O_10_, 436.4 Da) are two major active C-linked dihydrochalcones uniquely found in rooibos tea. Orientin and isoorientin (C_21_H_20_O_11_, 448.4 Da) are major C-glycosyl-containing flavones present widely in natural plants, including rooibos. The mean content of PPAG is similar to or more than ASP in FREs [10,11]. PPAG concentration in GREs is approximately 2%; however, that likely varies depending on batches and parts of plants, for example, stems vs. leaves [11,12,13].

Growing volumes of in vitro and in vivo data have suggested the potentially beneficial roles of rooibos tea extracts in glucose metabolism and associated complications, including oxidative stress, insulin resistance and diabetic myopathy [14]. In human subjects, a single dose of rooibos tea significantly reduced angiotensin-converting enzyme activity [15] and increased plasma antioxidant capacity [16], while chronic consumption of fermented rooibos tea improved markers for blood lipid levels and reduced those of oxidative stress [17]. However, no clinical trials have been performed to examine the effect of rooibos on diabetic parameters. Therefore, the aim of this study was to assess the effect of rooibos tea and associated polyphenols on blood glucose levels of diabetic rodent models by meta-analyzing the currently available studies and attempting to sort out the potential source of heterogeneity that may lead to the discrepancies in the current literature with subgroup and meta-regression analyses.

## 2. Results

### 2.1. Search Results

**Figure 1:**
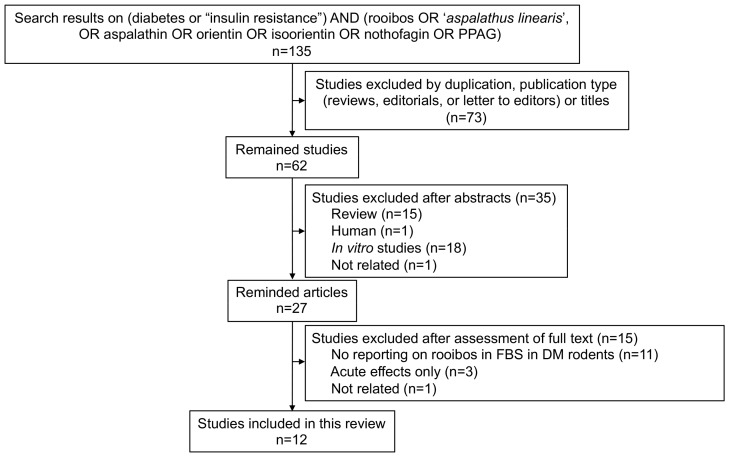
Flow diagram of literature search and selection process.

The flowchart of our literature search is shown in Figure 1. It resulted in a total of 135 articles (81 from Embase and 54 from PubMed). Upon removal of the duplicates and reviews of the titles and abstracts, 27 articles moved on to a full-text assessment. Of these, the majority of articles were excluded from the original meta-analysis because they failed to report blood glucose levels in DM rodent models treated with rooibos tea extracts or their associated polyphenols. Therefore, 12 studies were finally included in the meta-analyses [13,18,19,20,21,22,23,24,25,26,27,28]. There were no articles reporting the effect of either nothofagin, orientin, or quercetin-3-*O*-robinobioside alone on blood glucose levels in DM rodents.

### 2.2. Study Characteristics and Quality Assessment

The main characteristics of each included study are summarized in Table 1. Studies were generally published since 2005. The sample sizes ranged from 10 to 20 in each study. Of the included studies, animals are treated with FREs in two studies [18,28], GREs in one study [13], ASP in four studies [19,21,22,26], isoorientin in one study [25] and PPAG in four studies [20,23,24,27]. db/db mice were used in three studies, ob/ob mice in one study, diet-induced obese insulin-resistant (OBIR) rodents (in two studies one in rats and one in mice) and KK-Ay mice in one study and streptozotocin (STZ)-induced DM rodent models in five studies (three in rats and two in mice). Six studies used plasma for blood glucose measurement, one used serum and five used whole blood samples. Nine studies assessed glucose levels using fasting blood samples and three using non-fasting samples. 

The detailed quality assessment of each study is shown in Table S1. The study quality was fair in general with the risk of bias judged to be low to medium.

### 2.3. Effect of Rooibos Tea Extracts and Associated Polyphenols on Blood Glucose Levels in DM Rodent Models

**Figure 2:**
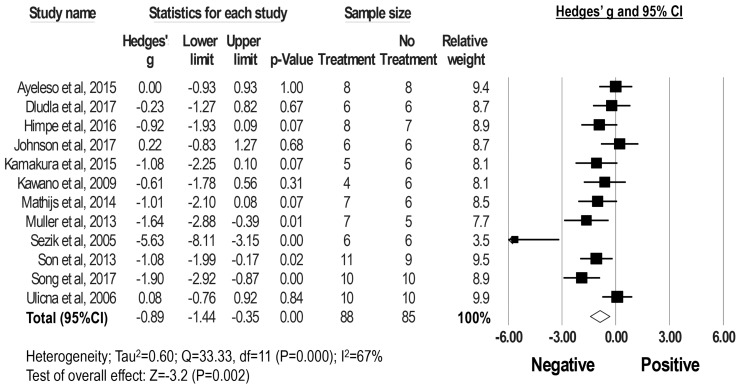
Flow diagram of literature search and selection process. Meta-analysis of Hedges’ g of blood glucose levels in DM rodents with and without treatment of rooibos extracts or associated major phenolic compounds. Summary estimates were analyzed using a random-effects model. CI, confidence interval.

Twelve studies from 12 articles enrolling 88 diabetic male rodents treated with rooibos extracts or their major polyphenol compounds, ASP, PPAG and isoorientin and 85 diabetic control males treated with vehicles reported their blood glucose levels and were included in this meta-analysis (Figure 2). Four studies showed that treatment with polyphenols, ASP, PPAG and isoorientin, significantly reduced blood glucose levels in DM rodent models [24,25,26,27]; eight studies did not observe any significant effects. By pooling all those studies using a random-effects model, results revealed that rooibos tea extracts or associated polyphenols reduce blood glucose levels in DM rodents (g = −0.89, 95% CI −1.44 to −0.35; *I^2^* = 67%, *p* < 0.001) (Figure 2). To determine the influence of each study on the overall result, the stability of results was next evaluated using a leave-one-out strategy. Upon removal of each individual study, all the re-pooled summary estimates remained unchanged compared with the primary estimates with the effect sizes ranging from −1.00 (95% CI, −1.57 to −0.42) to −0.70 (95% CI, −1.12 to −0.28).

**Figure 3:**
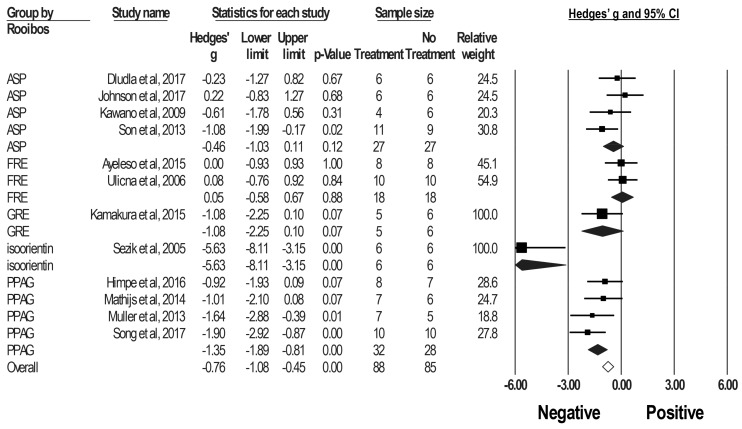
Subgroup analysis for Hedges’ g of blood glucose levels in DM rodents treated with various types of rooibos extracts and major phenolic compounds or vehicles. Summary estimates were analyzed using a random-effects model. CI, confidence interval.

Subgroup analyses suggested that PPAG and isoorientin have significantly reduced blood glucose levels in DM rodents (PPAG, g = −1.35, 95% CI: −1.89 to −0.81. isoorientin, g = −5.63, 95% CI: −8.11 to −3.15); however, FRE, GRE and ASP have little, if any, effect (Figure 3). Blood glucose levels are not influenced by type of rodent (mice vs. rats), blood sample (plasma, serum, vs. whole blood), or the blood sampling time point (non-fasting vs. fasting) (Table 2).

### 2.4. Meta-Regression Analyses

**Figure 4:**
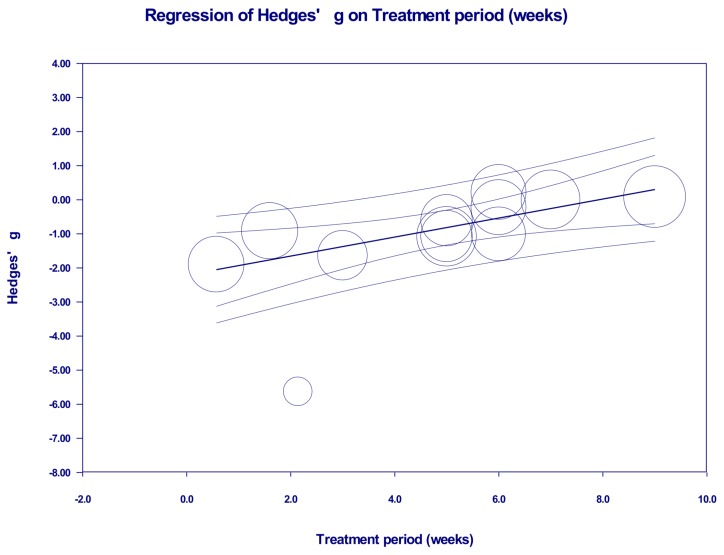
Meta-regression analysis for Hedges’ g of blood glucose levels and treatment period in DM rodents treated with or without rooibos extracts or major phenolic compounds. Summary estimates were analyzed using a random-effects model.

Univariate meta-regression analyses were performed next. Type of rooibos tea extracts and polyphenols is found to be a significant covariate to explain approximately 100% of between-study variance (*R^2^* = 1.0). The treatment period is also, at least in part, responsible for between-study variance (coefficient = 0.28, 95% CI: 0.11 to 0.45, *p* < 0.002, *R^2^* = 0.64) (Figure 4). Moreover, the analyses also showed that type of rodents, DM models, or blood samples and blood sampling time point do not affect the variance (*R^2^* = 0.00), confirming the results of subgroup analyses described above (Table 2).

### 2.5. Publication Bias

**Figure 5:**
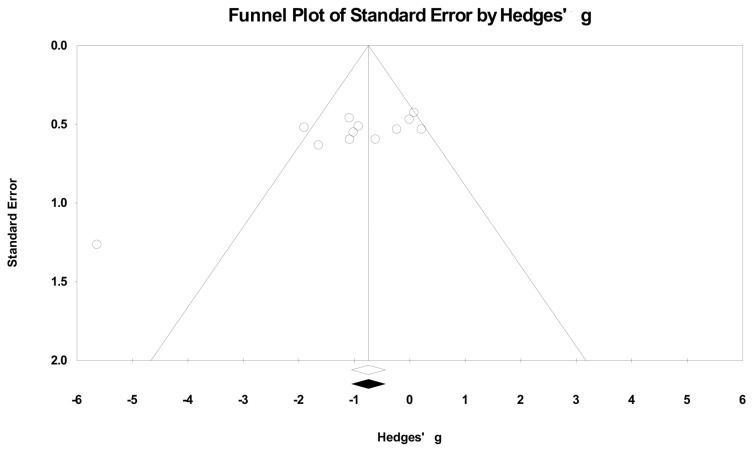
Funnel plots of standard error by Hedges’ g of blood glucose levels in DM rodents treated with or without rooibos extracts or major phenolic compounds. Open and closed diamond indicates the imputed summary estimates before and after Duval and Tweedie’s Trim and Fill adjustment (random-effects models), respectively. No imputed studies were found in re-displayed funnel plots by Duval and Tweedie’s Trim and Fill analysis.

No significant evidence of publication bias was observed in the analyses of the effect of rooibos tea extracts and polyphenols on blood glucose levels as indicated by funnel plots. Moreover, there were no imputed studies found in re-displayed funnel plots after Duval and Tweedie’s Trim and Fill adjustment [29,30] (Figure 5).

## 3. Discussion

### 3.1. Main Findings

To the best of our knowledge, this study is the first meta-analysis that summarizes the evidence for a possible beneficial role of rooibos extracts and associated polyphenols in blood glucose levels of DM rodent models. We showed that elevated blood glucose levels of DM rodents are significantly reduced by intake of FREs, GREs, and their major phenolic compounds compared with those of DM controls. This association was largely influenced by type of rooibos extracts and their polyphenols and, at least partly, by treatment period in the subgroup and meta-regression analyses, respectively. PPAG significantly reduced blood glucose levels in DM mice or rats; however, FREs, GREs, or ASP failed to demonstrate the similar effects. The chronical treatment with rooibos extracts or their major polyphenols was thus likely to lose the beneficial effect on reduced glucose levels over time. Furthermore, types of rodents (mice or rats), DM models (db/db, ob/ob, KK-Ay, OBIR, or STZ-induced) and blood samples (plasma, serum, or whole blood), or blood sampling time point (fasting or non-fasting) did not markedly influence the association.

### 3.2. Interpretation

#### 3.2.1. Structures and Pharmacological Properties of Phenolic Compounds Rich in Rooibos Extracts

**Figure 6:**
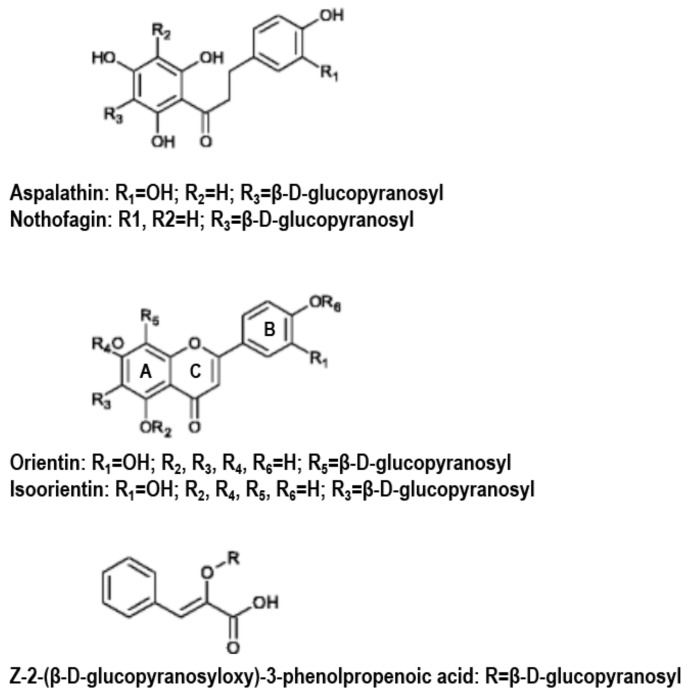
Structures of major flavonoids in rooibos extracts [14] Z-2-(β-d-glucopyranosyloxy)-3-phenylpropenoic acid, PPAG.

The structures of different classes of flavonoids present in the rooibos extracts are shown in Figure 6.

Two anti-diabetic pharmacological properties are known to be present in rooibos extracts. First, α-glucosidase inhibitors are oral anti-diabetic drugs used for treatment of T2DM by preventing glucose absorption in intestine and thus postprandial hyperglycemia. Muller et al. detected stronger inhibition of α-glucosidase activity in GREs. Further HPLC-based assay confirmed that α-glucosidase inhibitory activity corresponded with the retention time of ASP (Figure 6, top) [12]. Moreover, various C-glucoside flavones detected in rooibos extracts exhibited stronger α-glucosidase inhibition activity than acarbose, one of the most potent α-glucosidase inhibitors; the inhibitory activity decreases in the order isoorientin ≥ orientin ≥ isovitrexin ≥ vitrexin. The C-3 hydroxylation of the B-ring of flavones was suggested to be critical for the inhibition of α-glucosidase activity in flavones [31] (Figure 6, middle). Flavonols in rooibos extracts, isoquercitrin and rutin, also showed similar but much weaker α-glucosidase inhibitory activity than acarbose [32]. Thus, the glucose lowering effects of rooibos extracts could be due, at least partly, to the α-glucosidase inhibition activity of their contained flavonoids. Second, inhibition of glucose reabsorption in the kidney became a strategy for lowering blood glucose levels in T2DM [33]. The renal glomerulus filters approximately 160 g of glucose per day, 98% of which is then reabsorbed primarily in the proximal tubules of nephrons via sodium glucose co-transporters (SGLTs) [34]. Among six known SGLTs in human, SGLT1 and 2 have been well studied. SGLT1 is located in the small intestine, heart and kidney with an affinity for both glucose and galactose [35]; while, SGLT2 is localized only in the kidney with the high selectivity for glucose [36,37]. Therefore, chemical compounds with selective inhibition of SGLT2 over SGLT1 and a Glut family, another family of glucose transporters, would be ideal drug targets against T2DM. Phlorizin, a natural glucosylated dihydrochalcone present in the bark of apple trees, is the first reported SGLT inhibitor [38]. In the search of new selective drug targets for SGLT2, C-glucoside dihydrochalcones were examined; a line of research demonstrated the anti-SGLT2 activity in ASP and nothofagin, two major C-glucoside dihydrochalcone in rooibos [39,40]. Thus, the anti-SGLT2 property in ASP and nothofagin in rooibos extracts might also play a critical role in their in vivo anti-diabetic action.

#### 3.2.2. FREs, GREs and Major Phenolic Compounds in Rooibos in DM Rodent Models

FREs were shown to reduce DM-mediated H_2_O_2_- and ischemia-induced oxidative stress in STZ-injected DM rats. Aqueous and alkaline extracts of fermented rooibos tea were reported to significantly lower levels of oxidative stress markers, advanced glycation end products (AGEs) in plasma and advanced lipoxidation end products, malondialdehyde, in plasma, liver and lends of STZ-induced DM rats; the extracts also slightly, but not significantly, reduced advanced oxidation protein products [28]. Moreover, FREs increased oxygen radical absorbance capacity, superoxide dismutase and thiobarbituric acid reactive substances in STZ-induced DM rats [41]. However, FREs alone did not significantly improve plasma glucose and lipid profiles in DM rats [28]. Collectively, FREs might reduce oxidative stress in STZ-induced DM rats. However, consistent with the present meta-analysis, FREs alone are unlikely to significantly improve plasma glucose and lipid profiles, at least, in STZ-induced DM rats as reported in control rats [18,42].

In our subgroup analysis which included the solo study using KK-A^y^ mice, the chronic treatment with GREs for more than 3 days showed a trend but failed to exhibit significant beneficial effects on their blood glucose levels. However, it has been reported that acute and sub-chronical oral administration of GREs significantly lowered glucose levels in some DM rodent models [12]. The underlying mechanisms by which acute GREs reduce glucose levels could be that GREs stimulate glucose uptake in muscle [12,13] and liver cells [12], at least in part, by increasing phosphorylation of 5′-adenosine monophosphate-activated protein kinase (AMPK) and serine/threonine kinase (Akt), which then promotes translocation of glucose transporter 4 (Glut4) to the plasma membrane [13]. GREs also reduced AGE- and H_2_O_2_-induced oxidative stress in pancreatic β-cells [13]. Moreover, GREs showed to reverse the palmitate-induced insulin resistance and suppress inflammatory pathway by inhibiting palmitate-mediated nuclear factor-κB activation in 3T3-L1 adipocytes [43]. In summary, GREs have demonstrated more effective, yet not significant in this study, anti-diabetic potentials than FREs; GREs might reduce plasma glucose levels in a DM rodent model likely through mechanisms involving multi-organ systems such as liver, muscle, pancreas and adipose tissue.

The total antioxidant activity of GREs was positively associated with its ASP content [44]; therefore, several research examined how ASP alone could modulate glucose metabolism. A study first compared effects of GREs and ASP alone on glucose metabolism in STZ-induced DM rats [12]. The result suggests that GREs are more effective than ASP alone in glucose lowering effect in the DM mice, which is compatible with our subgroup analysis (ASP; −0.46 (−1.03 to 0.11) *p* = 0.12 vs. GRE; −1.08 (−2.25 to 0.10) *p* = 0.07). This was likely because GREs contain other polyphenols such as rutin [45,46,47,48,49,50] and vitexin/isovitexin [51,52], which previously showed glucose lowering effects in STZ-induced DM rats. ASP suppressed the increased fasting blood sugar levels and/or improved glucose tolerance in two T2DM and obese mouse models, db/db [22] and ob/ob [26] mice. The consequent in vitro studies demonstrated that these effects were presumably due, at least in part, to an ASP-mediated dose-dependent increase in glucose uptake in the muscle cells; ASP enhanced phosphorylation of AMPK and promoted Glut4 translocation to the plasma membrane [13,22,26]. ASP also increased insulin secretion [22] and reduced AGE-mediated rise in reactive oxygen species, a marker for oxidative stress, [26] in pancreatic β-cells. Collectively, ASP exhibited two anti-diabetic pharmacological properties, inhibition of both α-glucosidase and SGLT2 activities and glucose lowering effects presumably targeting muscle cells in some T2DM mouse models, however, the present study failed to display a marked reduction of blood glucose levels by the administration of ASP alone to DM rodents.

Analysis of infusions prepared from various production batches of fermented rooibos demonstrated that PPAG is one of the major monomeric phenolic compounds present at similar concentrations to ASP (Figure 6, bottom) [53]. Biological studies showed that PPAG delayed the onset of diabetes by modifying cell death and necrosis, but not by increasing cell proliferation or its ability for DNA damage/repair, of pancreatic β-cells in STZ-induced DM mice [20]. Moreover, PPAG has been recently shown to improve fasting blood glucose levels, glucose tolerance, insulin levels and insulin-resistance in OBIR rats [24]. The effects were presumably through increased expression of glucokinase, Glut 1 and 2, insulin receptor, peroxisome proliferator-activated receptor α and suppressor of cytokine signaling 3 in the liver and through reduced apoptosis or neogenesis of pancreatic β-cells [24]. Therefore, these results suggest that PPAG could be a significant modulator for glucose metabolism in the liver and pancreas in the rodent DM models and our meta-analysis supports a significant role of PPAG in regulation of blood glucose levels.

The role of isoorientin in STZ-induced DM mice was investigated as the main constituent of *Cecropia obtusifolia* or *Gentiana olivieri*, plants found in Central America (Columbia, Costa Rica, Mexico and Panama) or Asia, respectively [25,54]. Aqueous extracts of *Cecropia obtusifolia* was described for use of the treatment of diabetes in mice and rabbits [55,56]. Dried flowering herbs of *Gentiana olivieri* in water was used to lower blood glucose levels of T2DM patients [57]. The present meta-analysis based on a single study also suggested the significant glucose lowering effect of isoorientin in DM rodent models; this could be due to strong pharmacological ability to inhibit α-glucosidase activity as mentioned above. However, the strength of meta-analysis with one study using a dozen rats is quite low and more future studies will be thus necessary to draw a certain conclusion regarding an effect of isoorientin on blood glucose levels.

#### 3.2.3. Strength and Limitations

The primary strength of this meta-analysis is the inclusion of relatively large number of DM mouse and rat models and focusing on an effect of rooibos on their blood glucose levels. We also systematically assessed various cofounding factors. This meta-analysis has also several limitations. First, although a broad literature search was applied using two electronic databases, the number of articles assessing an effect of GREs and isoorientin on blood glucose levels in DM rodents was quite small and the language restriction and the exclusion of ambiguous literature might increase the risk of publication bias. Second, some evidence of heterogeneity existed in our meta-analysis although this could be mostly explained by the pre-specified variables, type of tested rooibos and related polyphenols as well as treatment period. This heterogeneity may also potentially weaken the robustness of our findings. Third, all the included studies used male rodents, the outcome could be different when studies include females. Forth, STZ-induced DM rodent models were generated after grouping for diet modulation in three included studies [20,27,28]. There exists, at least, a slight possibility that the investigators’ technical skill for STZ injection could directly or indirectly influenced the experimental outcome of blood glucose levels.

## 4. Materials and Methods

### 4.1. Data Sources and Search Strategies

A comprehensive literature search of the electronic databases PubMed and Embase for the period between 1 January 1962 and 28 February 2018 was conducted using the keywords (“*Aspalathus linearis*” or rooibos or aspalathin or nothofagin or PPAG or orientin or isoorientin or quercetin-3-*O*-robinobioside) and (diabetes or “insulin resistance”). In addition, the reference lists of the retrieved articles were manually searched to ensure that no relevant articles had been missed.

### 4.2. Inclusion and Exclusion Criteria

Peer-reviewed articles in the English language were eligible for inclusion when they fulfilled the following inclusion criteria: (i) studies used diabetic rodent models with or without treatment with fermented or green rooibos extracts (FRE and GRE, respectively), PPAG, ASP, nothofagin, orientin, isoorientin, or quercetin-3-*O*-robinobioside for more than 3 days; (ii) they also reported the blood glucose levels of the DM rodents at the end of the treatment period. As the polyphenols associated with fermented and green rooibos teas, PPAG, ASP, nothofagin, orientin, isoorientin and quercetin-3-*O*-robinobioside are included because they were previously reported to be contained more than 10% *w/w* of total phenolic compounds either in FREs or GREs. Studies were excluded if they are reviews, commentaries, editorials, letters, conference abstracts, duplicates, not in English, or not studied on blood glucose levels with the treatment for longer than 3 days in rodent DM models. Unpublished research was not sought. This meta-analysis was strictly conducted according to the PRISMA guidelines (Table S2).

### 4.3. Data Extraction and Quality Assessment

Titles and abstracts of retrieved publications were screened initially for potentially eligible studies, which were subsequently evaluated by full-text review. Data were collected by three authors (M.S., N.N. and M.S.) in an independent manner using pre-designed standardized data extraction form; which includes type of rooibos extracts or associated compounds, dose, method and period of administration, blood glucose levels, baseline age, sex, type of rodent DM models and their relevant controls. Study quality was assessed by the Cochrane Collaboration “Risk of Bias” Tool [58]. The risk of bias for each quality variable in each criterion was assessed by 2 authors in an independent manner and judged as “low”, “unclear”, “high”, or “not applicable (N/A)” based on its description in each included study. Any disagreements in any phase were resolved by discussion until consensus was achieved.

### 4.4. Data Synthesis and Analysis

Continuous variables were presented as means ± standard deviation (SD). For studies reporting the standard errors of means (SEs), the corresponding SDs were calculated by multiplying by the square root of the respective sample size. For studies providing glucose levels in mmol/L, these levels were converted into mg/dL using the conversion table offered by the Joslin Diabetic Center at http://www.joslin.org/info/conversion_table_for_blood_glucose_monitoring.html. For studies reporting more than one measure of blood glucose levels, the levels after the longest period of treatment with rooibos extracts or associated compounds and at the treatment dose which gave the most robust difference in blood glucose levels between the two DM rodent groups were selected and included in the primary meta-analyses.

Standardized mean difference, Hedges’ g, transformation was used to calculate the related statistics including variance and 95% CIs of each study and the summary effect size generated in the meta-analyses and publication bias assessment. The random-effects model was chosen in this study because it is more conservative and incorporates better between-study variability. Heterogeneity was assessed using *I^2^* statistics with its value ≥50% interpreted as evidence of substantial heterogeneity [58].

Subgroup and meta-regression analyses were performed based on type of rooibos extracts and their major polyphenols (FRE, GRE, ASP, PPAG, or isoorientin), type of blood samples (whole blood, plasma, or serum), blood sampling time point (fasting vs. ad libitum (ad lib.)), types of rodents (mice vs. rats) and DM models (db/db, ob/ob, KK-Ay, or STZ-induced) and treatment period to examine their influence to the outcome estimates. Sensitivity analyses were used to evaluate the robustness of the outcome estimates mainly by removing one study at a time with a repeat of the primary meta-analyses. Publication bias was assessed by funnel plots with Duval and Tweedie’s Trim and Fill analysis (random-effect models). All the statistical analyses were carried out using Comprehensive Meta-Analysis 3.0 (Biostat, Englewood, NJ, USA) and Review Manager (Version 5.3, the Nordic Cochrane Center, Copenhagen, Denmark) software.

## 5. Conclusions

The present meta-analyses demonstrated that blood glucose levels were significantly reduced in diabetic rodent models treated with PPAG, a rooibos-associated phenolic compound. Some sporadic case studies reported severe yet reversible adverse effects of rooibos tea on liver in humans [59,60]. Thus, further clinical studies would be needed to establish the safe and practical use of the rooibos tea for prevention and treatment of diabetes and its associated complications in humans in the future. Finally, it would have a profound impact on an increasing number of pre-diabetic patients worldwide, in particular, if herbal teas such as rooibos could be developed as natural nutraceuticals for prevention or delayed onset of diabetes.

## Figures and Tables

**Table 1:** Characteristics of included studies in the meta-analysis.

Authors (Year)	Rooibos or Poly-phenols	Dose, Route	Duration	Animal Models	Total *n* (T/no-T)	Age or Weight at a Baseline	Diet	Fasting or ad Lib.	Blood Sample
Ayeleso A et al., (2015) [18]	FRE	2 g/100 mL boiling water. As drinking water	7 w	STZ-induced DM rats (50 mg/kg i.m.)	16 (8/8)	176–255 g	Control	Overnight fasting	Plasma
Dludla PV et al., (2017) [19]	ASP (98%)	13 or 130 mg/kg BW via daily oral gavage	6 w	db/db mice	12 (6/6)	9 w	Control	16-h fasting	Plasma
Himpe E et al., (2016) [20]	PPAG (99%)	10 mg/kg BW via daily oral gavage.	11 d	STZ-induced DM mice (200 mg/kg i.p.)	15 (8/7)	9–11 w, approx. 25 g	Control	Ad lib.	Whole blood
Johnson R et al., (2017) [21]	ASP (98%)	13 or 130 mg/kg/day via daily oral gavage	6 w	db/db mice	12 (6/6)	9 w	Control	4-h fasting	Plasma
Kamakura R et al., (2015) [13]	GRE (6.62% ASP)	Add to diet at 0.3% and then 0.6%.	5 w	KK-Ay mice	11 (5/6)	4 w	Control	3-h fasting	Whole blood
Kawano A et al., (2009) [22]	ASP (98.5%)	Added to diet at 0.2%	5 w	db/db mice	10 (4/6)	6 w	Control	4-h fasting	Whole blood
Mathijs I et al., (2014) [23]	PPAG (99%)	10 mg/kg BW via daily oral gavage	6 w	OBIR mice	13 (7/6)	15 w	High fat and fructose	Fasting	Whole blood
Muller CJ et al., (2013) [24]	PPAG (99%)	0.3–3 mg/kg BW via daily oral gavage	3 w	OBIR rats	12 (7/5)	24 w	High fat and sucrose	4-h fasting	Plasma
Sezik E et al., (2005) [25]	isoorientin	15 or 30 mg/kg BW/d via daily oral gavage	15 d	STZ-induced DM rats (55mg/kg i.p.)	12 (6/6)	200–250 g	Control	18–20 h fasting	Whole blood
Son MJ et al., (2013) [26]	ASP	0.1% dietary supplement	5 w	ob/ob mice	20 (11/9)	6 w	Control	3-h fasting	Serum
Song I et al., (2017) [27]	PPAG	A dose of 10 mg/kg BW via daily oral gavage	4 d	STZ-induced DM mice (200 mg/kg i.p.)	20 (10/10)	9–11 w, approx. 25 g	Control	Ad lib.	Whole blood
Ulicna O et al., (2006) [28]	FRE	2.5 g/1L of boiling water, 5 mL/kg BW/d via gavage	9 w	STZ-induced DM rats (45 mg/kg i.v.)	20 (10/10)	290–340 g	Control	Ad lib.	Plasma

FRE, fermented rooibos extract; BW, body weight; STZ, streptozotocin; GRE, green rooibos extract; ASP, aspalathin; PPAG, phenylpyruvic acid-2-*O*-glucoside; i.v., intravenous, i.m., intramuscular; i.p., intraperitoneal. T, treatment; no-T, non-treatment.

**Table 2:** Subgroup analyses.

Subgroups	Effect Size					Heterogeneity (*I^2^*)
	No. of Studies	*g*	95% CI		*P*-value	
**Rooibos and Polyphenols**						
FRE	2	0.05	−0.58	0.67	0.88	<0.001
GRE	1	−1.08	−2.25	0.10	0.07	<0.001
ASP	4	−0.46	−1.03	0.11	0.12	18.12
PPAG	4	−1.35	−1.89	−0.81	<0.001	<0.001
Isoorientin	1	−5.63	−8.11	−3.15	<0.001	<0.001
**DM rodent models**						
db/db	3	−0.18	−0.80	0.45	0.02	<0.001
ob/ob	1	−1.08	−1.99	−0.17	0.58	<0.001
KK-Ay	1	−1.08	−2.25	0.10	0.07	<0.001
OBIR	2	−1.28	−2.10	−0.46	0.002	<0.001
STZ	5	−1.29	−2.54	−0.05	0.04	84.65
**Rodent**						
Mice	8	−0.84	−1.28	−0.39	<0.001	30.09
Rats	4	−1.41	−3.03	0.22	0.09	86.70
**Blood sample**						
Plasma	6	−0.54	−1.26	0.19	0.15	67.42
Serum	1	−1.08	−1.99	−0.17	0.02	<0.001
Whole blood	5	−1.43	−2.47	−0.39	0.01	70.58
**Sampling time point**						
Non-fasting	3	−0.88	−2.03	0.27	0.134	77.02
Fasting (>3h)	9	−0.91	−1.58	−0.24	0.007	67.51

## Supplementary Materials

The following are available online. Table S1: Risk of Bias; Table S2: PRISMA checklist.
